# Prognostic value of platelet to lymphocyte ratio in hepatocellular carcinoma: a meta-analysis

**DOI:** 10.1038/srep35378

**Published:** 2016-10-14

**Authors:** Wencong Ma, Ping Zhang, Jun Qi, Litong Gu, Mingcui Zang, Haochen Yao, Xiaoju Shi, Chunli Wang, Ying Jiang

**Affiliations:** 1 Department of Hepatobiliary Surgery, First Hospital of Jilin University, Changchun 130021, China; 2 Department of Dermatology, First Hospital of Jilin University, Changchun 130021, China

## Abstract

This study was designed to evaluate the prognostic value of platelet to lymphocyte ratio (PLR) in hepatocellular carcinoma (HCC). A comprehensive literature search for relevant studies was performed in Web of science, Embase and Pubmed. A total of nine studies with 2017 patients were included in this meta-analysis, and combined hazard ratio (HR) or odds ratio (OR) and 95% confidence intervals (95%CIs) were served as effect measures. Pooled results showed that elevated PLR was associated with poor overall survival (OS) (HR = 1.63, 95%CI: 1.42–1.88, p = 0.000; I^2^ = 0.0%, P_h_ = 0.637) and poor disease-free survival (DFS)/recurrence-free survival (RFS) (HR=1.32, 95%CI: 1.15–1.52, p = 0.000; I^2^ = 19.3%, P_h_ = 0.287) in HCC patients. In addition, high PLR was not significantly correlated with the presence of vascular invasion, tumor multifocality, poor tumor grade or high level of serum AFP (>400 ng/ml). In conclusion, elevated PLR indicated a poor prognosis for patients with HCC. PLR may be a reliable, easily-obtained, and low cost biomarker with prognostic potential for HCC.

Hepatocellular carcinoma (HCC) is one of the most common and aggressive malignancies. Meanwhile, it is the third leading cause of cancer-related deaths across the world^1^. Despite the major treatment methods of HCC, including surgical resection, liver transplantation, radiofrequency ablation, transarterial chemoembolization (TACE) and molecular therapy have achieved significant improvements, the prognosis of the patients remains unsatisfactory due to the distant metastasis and high fatality^2^. Several criteria have been proposed to predict patient prognosis, like functional liver reserve, Tumor Node Metastasis (TNM), Barcelona Clinic Liver Cancer (BCLC) score and Cancer of the Liver Italian Program (CLIP) staging score. However, since these criteria are cumbersome, they are rarely used in routine clinical practice. Thus, the identification of an efficient, simple and easily-obtained prognostic biomarker, especially serum biomarkers for prognosis and recurrence of HCC, is essential.

Recently, a variety of inflammatory indices such as C-reactive protein (CRP), neutrophil to lymphocyte ratio (NLR), platelet to lymphocyte ratio (PLR), and modified Glasgow prognostic score (mGPS) have demonstrated their prognostic value in multiple cancers^3^,^4^,^5^,^6^. Among these markers, elevated PLR was identified as an unfavourable prognostic factor in various cancers such as colorectal cancer^7^, breast cancer^8^ and gastric cancer^9^. The prognostic value of PLR in HCC has also been investigated^10^,^11^,^12^.

Nevertheless, conflicting data have emerged concerning the prognostic value of PLR to predict disease progression and overall survival (OS) in HCC. We therefore collected the available publications and conducted this meta-analysis to evaluate the impact of PLR for OS and disease-free survival (DFS)/recurrence-free survival (RFS) in HCC. In addition, the correlation between PLR and patients’ clinicopathological features was also examined.

## Results

### Selection and characteristics of studies

**Figure 1:**
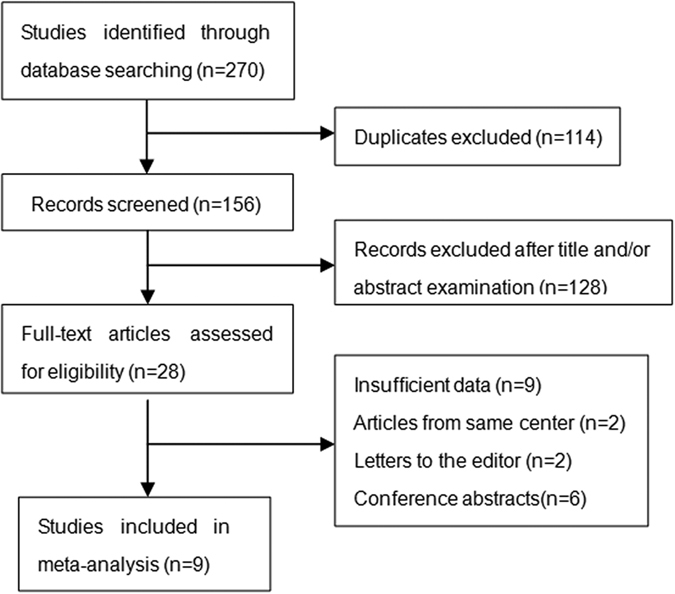
Flow diagram of the study selection procedure.

The literature searching progress was showed in Fig. 1. A total of 9^11^,^13^,^14^,^15^,^16^,^17^,^18^,^19^,^20^ eligible studies published between 2012 and 2016 with 2017 patients were identified. The basic characteristics of the original studies were presented in Table 1. Of these studies, two studies^11^,^16^ were reported by the same center,but according to the inclusion and exclusion criteria, the patients included in these two studies were not overlapping. Five studies^11^,^14^,^16^,^17^,^18^ were conducted in China, the other four studies^13^,^15^,^19^,^20^ were conducted in Hong Kong China, USA, UK and Singapore, respectively. Surgical resection as initial treatment for HCC was reported in 6 studies^13^,^14^,^16^,^18^,^19^,^20^. Mix treatment (locoregional, systemic treatments and supportive care)^15^ and TACE^11^ were reported in one study.respectively. One study^17^ just demonstrated that all patients were not receiving sorafenib as systemic treatment instead of providing details of the treatment. Seven studies^11^,^14^,^16^,^17^,^18^,^19^,^20^ defined OS as the length of time from initial treatment to death or last follow up, while the other two studies^13^,^15^ generally described OS as overall survival. One study^14^ gave the definition of DFS as the time from treatment initiation until disease progression or death. Three studies^16^,^18^,^20^ defined DFS as the duration of time between the date of treatment and the date of first recurrence. One study^19^ provided the definition of RFS as the time from initial treatment to first recurrence and death without disease was censored. One study^13^ generally described RFS as recurrence-free survival. Sample sizes ranged from 80 to 367. The prognostic value of PLR for OS was reported or estimated in all studies, whereas the prognostic significance of PLR in DFS/RFS was only provided in six studies^13^,^14^,^16^,^18^,^19^,^20^ in which the included patients undertook surgical resection as initial treatment. The cut-off values used for PLR in these studies were determined by different methods and ranged from 112.3 to 300. The scores of study quality assessed by Newcastle-Ottawa quality assessment scale ranged from 5 to 8(with a mean of 7). A high value indicated better methodology.

### PLR and OS in HCC

**Figure 2:**
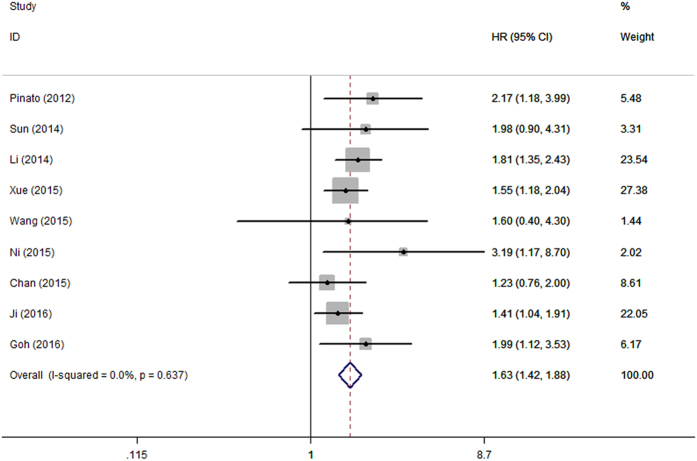
Forrest plot of hazard ratio (HR) for the association of PLR with OS in patients with HCC.

Pooled data from all the nine studies revealed that elevated PLR was significantly associated with poor OS with a pooled HR of 1.63 (95%CI: 1.42–1.88, p = 0.000; Fig. 2), and without significant heterogeneity in the data (I^2^ = 0.0%, P_h_ = 0.637).

Subgroup analysis of six studies^13^,^14^,^16^,^18^,^19^,^20^ with 1371 patients who underwent surgery only showed that elevated PLR predicted poor OS (HR = 1.54, 95%CI: 1.24–1.91, p = 0.000; I^2^ = 0.0%, P_h_ = 0.499).

### PLR and DFS/FRS in HCC

**Figure 3:**
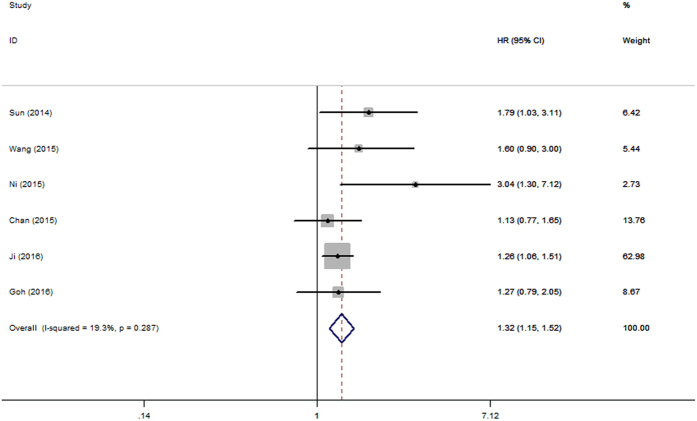
Forrest plot of hazard ratio (HR) for the association of PLR with DFS/RFS in patients with HCC.

There were six studies^13^,^14^,^16^,^18^,^19^,^20^ with 1371 patients investigated the association between PLR and DFS/RFS. The combined data revealed that elevated PLR was correlated with shortened DFS/RFS (HR = 1.32, 95%CI: 1.15–1.52, p = 0.000; I^2^ = 19.3%, P_h_ = 0.287; Fig. 3).

### Associations between PLR and clinicopathologic features in HCC

The associations between PLR and clinicopathologic parameters were summarized in Table 2. Four studies^11^,^14^,^15^,^16^ reported data about the correlation between elevated PLR and high level of serum AFP (>400 ng/ml). Three studies suggested no correlation, while one study reported statistical significance. Pooled data from all the four studies did not support a correlation (OR = 1.24, 95%CI: 0.87–1.75, p = 0.229; I^2^ = 16.1%, P_h_ = 0.311). As for the other three clinicopathologic features: vascular invasion, tumor multifocality and poor tumor grade, combined data did not show statistical significance either.

### Sensitivity analysis

**Figure 4:**
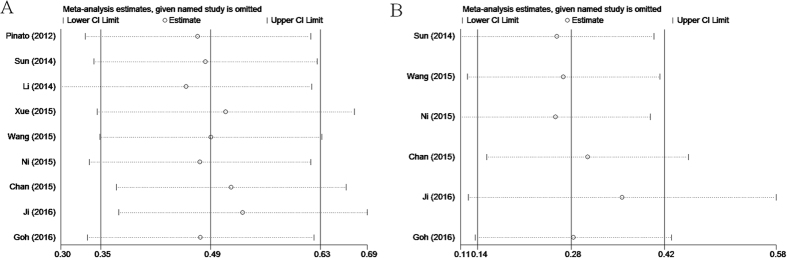
Sensitivity analysis on the relationship between PLR and (**A**) OS and (**B**) DFS/RFS in HCC.

Each individual study was omitted every time to estimate the influence of individual data sets on the pooled HR. The results showed that the corresponding HRs for OS and DFS/RFS were not markedly changed (Fig. 4), indicating the robustness of presented results.

### Publication bias

**Figure 5:**
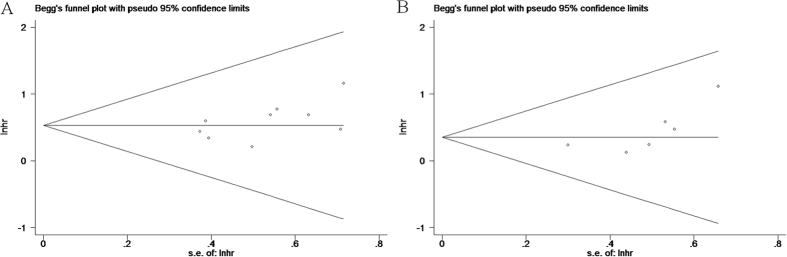
Begg’s funnel plot of publication bias test for (**A**) OS and (**B**) DFS/RFS in HCC.

The results of Begg’s test suggest no evidence of publication bias (p = 0.175 for OS and P = 0.060 for DFS/RFS, respectively)(Fig. 5).

## Discussion

In the present study, we mainly investigated the prognostic impact of pretreatment PLR on OS and DFS/RFS in patients with HCC by the method of meta-analysis. The pooled outcomes from nine primary studies with 2017 patients demonstrated that elevated PLR predicted poor OS (HR = 1.63, 95%CI: 1.42–1.88, p = 0.000; I^2^ = 0.0%, P_h_ = 0.637) and poor DFS/RFS (HR = 1.32, 95%CI: 1.15–1.52, p = 0.000; I^2^ = 19.3%, P_h_ = 0.287) in HCC. It should be noted that the result was not substantially changed in sensitivity analysis. Furthermore, stratified analysis revealed that elevated PLR was also significantly correlated with shortened OS(HR = 1.54, 95%CI: 1.24–1.91, p = 0.000; I^2^ = 0.0%, P_h_ = 0.499) in HCC patients treated by surgical resection.

The clinicopathologic features of HCC, especially tumor multifocality and vascular invasion were reported to be associated with the prognosis and survival of HCC^21^. In this circumstance, we conducted pooled analysis to evaluate the associations between pretreatment elevated PLR and clinicopathologic features in HCC. However, the result indicated that elevated PLR was not significantly associated with the presence of vascular invasion, tumor multifocality (satellite nodule), high level of serum AFP (>400 ng/ml) or poor tumor grade(Edmonson grade 3 or 4).

Accumulated evidence has showed that systematic inflammatory response plays an important role in tumor initiation and progression^22^,^23^. The exact mechanism is still unknown. While a large amount of studies demonstrated that the inflammation of microenvironment could influence the proliferation and survival of tumor cells^24^,^25^. Since most of the HCC patients are related to the chronic HBV or HCV infection, the patients would experience inflammation chronically. Platelets, a participant of inflammatory response, have been reported to protect tumor cells from natural killer-mediated lysis, thus supporting the tumor metastasis^26^. Additionally, the platelet-derived growth factor, basic fibroblast growth factor and hepatocyte growth factor could enhance tumor cells’ capability to metastasis^27^. Furthermore, thrombocytosis was shown to be an independent prognostic factor for epithelial ovarian cancer^28^. On the other hand, lymphocytes play a significant role in anti-tumor immune response. In HCC patients, increased infiltration of CD4+ T lymphocytes at the tumor margins has been reported to be associated with a lower recurrence rate and better prognosis^29^. However, Schreiber *et al*.^3^reported that tumor cells could reduce cytotoxic T lymphocyte (CTL) infiltration by secreting immunosuppressive cytokines such as vascular endothelial growth factor (VEGF), transforming growth factor–β (TGF-β), IL-10 and by consuming IL-2, a cytokine that is critical for maintaining CTL function. Thus, platelets and lymphocytes are tightly correlated with tumor progression. Previous meta-analyses^31^,^32^,^33^ have demonstrated the negative impact of elevated PLR on the prognosis of esophageal cancer and nonsmall cell lung cancer. Our study was the first study investigating the prognostic significance of PLR for HCC patients and the results were consistent with previous reports. Due to the convenience to obtain and low cost, the PLR could be a promising biomarker for clinical use.

There still existed several limitations of this study. First, HRs and 95%CIs were not directly provided in some studies, and we had to use Tierney’s method^34^ to calculate the value from supplied data. Second, due to the insufficient data to obtain or calculate HRs and 95%CIs of PLR for OS and DFS/RFS, none of studies concerning the prognostic value of PLR on HCC patients treated by liver transplantation were included in this meta-analysis. And this may limit the usefulness of PLR in clinical practice. Third, all of the included studies were retrospective and published in English. Finally, we analyzed the correlations between the elevated PLR and clinicopathological parameters of patients. However, for each clinicopathological feature, there were only 3 or 4 studies reporting the relevant information.

In conclusion, our results indicated that elevated pretreatment PLR might be an unfavorable prognostic factor for OS and DFS/RFS in patients with HCC, which could be useful in stratifying patients and determining individual treatment plan. However, considering the limitations listed above, more well-designed and large-scale investigations are warranted to better understand the value of PLR in the prognosis of HCC.

## Methods

### Search strategy

The following databases were systematically searched until April 2016 without time restrictions: Web of science, Embase and Pubmed. The search strategy was based on combination of following terms: “PLR”, ” platelet to lymphocyte ratio”, “platelet-lymphocyte ratio”, “hepatocellular carcinoma”, ”HCC”, “liver carcinoma”, ”liver cancer ”. Reports in English were eligible for inclusion. Reviews and reference lists in each identified publications were also manually retrieved for additional publications.

### Inclusion and exclusion criteria

The inclusion criteria were as follows: (1) HCC was diagnosed on pathology or the diagnostic criteria of the American Association for the Study of Liver Disease; (2) PLR was measured by serum-based methods before formal treatment; (3) HRs and 95%CIs for PLR in OS and/or DFS/RFS were described in the study or could be calculated from the supplied data.

The exclusion criteria were as follows: (1) reviews, conference abstract, letter, full text not available in English.; (2) overlapping or duplicate data; (3) did not provide the cut-off value for elevated PLR; (4) nonhuman studies.

### Data extraction and quality assessment

All data extractions were performed separately by two independent investigatoras (W.C.M. and Y.J.) and disagreements were resolved by joint discussion. The following data were recorded for each eligible study: family name of the first author, year of the publication, country of the origin, sample size, treatment methods, cut-off values of PLR, survival data and clinicopathologic parameters. The quality of the included studies was assessed according to the Newcastle-Ottawa Quality Assessment Scale (NOS) by two independent investigators (W.C.M. and Y.J.). The NOS consists of three aspects: selection (four points), comparability (two points), and outcome assessment (three points). NOS scores ≥6 were regarded as high-quality studies.

### Statistical Analysis

The impact of PLR on OS and DFS/RFS was measured by combined HRs and their 95% CIs which were directly extracted from each eligible articles or estimated according to the methods reported by Tierney *et al*.^3^ As for the impact of PLR on clinicopathologic features, pooled ORs and their 95% CIs were used. The Cochrane Q test and I^2^ statistic were used to assess the heterogeneity of the pooled results. Cochran Q test’s p value < 0.10 or I^2^ >50% was considered as large heterogeneity between studies and random effect model (DerSimonian Laird method) was performed to calculate the pooled HR and 95% CI. In other cases, fixed effects model (Mantel-Haenszel method) was adopted.

Sensitivity analysis was conducted by removing each study and recalculating the combined HRs. Begg’s test was used to evaluate the publication bias. All statistical analyses were performed using Stata 12 (Stata Corp., College Station, Texas). P < 0.05 was considered statistically significant.

## Additional Information

**How to cite this article**: Ma, W. *et al*. Prognostic value of platelet to lymphocyte ratio in hepatocellular carcinoma: a meta-analysis. *Sci. Rep.* **6**, 35378; doi: 10.1038/srep35378 (2016).

## Figures and Tables

**Table 1:** Baseline characteristics of all the included studies.

Study	Year	Country	Sample Size	Mean/median	Stage BCLC/TNM	PLR Cut-off	Treatment	Outcome	Hazard ratio	Follow-up (month)	NOS score
Pinato^18^	2012	UK	112	65	BCLC/A-D	300	Mix	OS	R	10(median)	7
Sun^17^	2014	China	80	47	TNM/I-III	151.8	Surgery	OS/DFS	E/R	NA	7
Li^20^	2014	China	243	57	BCLC/C.D	111.23	No sorafenib	OS	E	2.7(median)	6
Xue^15^	2015	China	291	53.05	BCLC/B.C	150	TACE	OS	R	9(median)	8
Wang^16^	2015	US	113	55.5	NA	118.5	Surgery	OS/RFS	R/R	NA	5
Ni^19^	2015	China	367	NA	BCLC/A-C	150	Surgery	OS/DFS	E/E	24(median)	6
Chan^23^	2015	Hong Kong	324	56.8	BCLC/0.A	150	Surgery	OS/DFS	R/R	44.6	8
Ji^21^	2016	China	321	51	TNM/I-III	115	Surgery	OS/DFS	R/E	NA	8
Goh^22^	2016	Singapore	166	66	NA	290	Surgery	OS/RFS	E/E	23(median)	8

BCLC: Barcelona Clinic Liver Cancer score; NA: not available; R: reported in article; E: estimated; OS: overall survival; DFS: disease-free survival; RFS: recurrence-free survival; NOS: Newcastle-Ottawa quality assessment scale.

**Table 2:** Associations between PLR and clinicopathologic features.

Clinicopathologic feature	Study	No. of patients	OR (95%CI)	P	Effects model	Heterogebeity	
						I^2^%	P_h_
AFP > 400 ng/ml	Pinato^18^, Sun^17^, Xue^15^, Ni^19^	843	1.24(0.87–1.75)	0.229	F	16.1	0.311
Vascular invasion	Xue^15^, Ni^19^, Goh^22^	824	1.03(0.70–1.53)	0.878	F	37.1	0.204
Tumor multifocality	Pinato^18^, Ni^19^, Goh^22^	643	1.10(0.58–2.05)	0.777	F	0	0.92
Poor tumor grade	Sun^17^, Ni^19^, Goh^22^	613	1.18(0.73–1.91)	0.493	F	0	0.925

OR: odds ratio; F: fixed-effects models; P_h_: p value of Q test for heterogeneity.
